# Identifying depression with mixed features: the potential value of eye-tracking features

**DOI:** 10.3389/fneur.2025.1555630

**Published:** 2025-03-19

**Authors:** Xing-Chang Liu, Ming Chen, Yu-Jia Ji, Hong-Bei Chen, Yu-Qiao Lin, Zhen Xiao, Qiao-Yan Guan, Wan-Qi Ou, Yue-Ya Wang, Qiao-Ling Xiao, Xin-Cheng-Cheng Huang, Ji-Fan Zhang, Ye-Kai Huang, Qian-Ting Yu, Mei-Jun Jiang

**Affiliations:** ^1^Guangdong Mental Health Center, Guangdong Provincial People’s Hospital (Guangdong Academy of Medical Sciences), Southern Medical University, Guangzhou, Guangdong, China; ^2^Department of Psychiatry, The First Affiliated Hospital of Guangzhou Medical University, Guangzhou, Guangdong, China; ^3^The Second School of Clinical Medicine, Southern Medical University, Guangzhou, Guangdong, China

**Keywords:** depression, mixed features, eye-tracking, machine learning, diagnosis

## Abstract

**Objectives:**

To investigate the utility of eye-tracking features as a neurobiological marker for identifying depression with mixed features (DMF), a psychiatric disorder characterized by the presence of depressive symptoms alongside subsyndromal manic features, thereby complicating both diagnosis and therapeutic intervention.

**Methods:**

A total of 93 participants were included, comprising 41 patients with major depressive disorder (MDD), of whom 20 were classified as DMF, and 52 healthy controls (HC). Eye-tracking features were collected using an infrared-based device, and participants were evaluated using clinical scales including the Montgomery-Åsberg Depression Rating Scale (MADRS), Young Mania Rating Scale (YMRS), and Brief Psychiatric Rating Scale (BPRS). Performance of extreme gradient boosting (XGBoost) model based on demographic and clinical characteristics was compared with that of the model created after adding ocular movement data.

**Results:**

Significant differences were observed in certain eye-tracking features between DMF, MDD, and HC, particularly in orienting saccades and overlapping saccades. Incorporating eye-tracking features into the XGBoost model enhanced the predictive accuracy for DMF, as evidenced by an increase in the area under the curve (AUC) from 0.571 to 0.679 (*p* < 0.05), representing an 18.9% improvement. This suggests a notable enhancement in the model’s ability to distinguish DMF from other groups. The velocity of overlapping saccades and task completion time during free viewing were identified as significant predictive factors.

**Conclusion:**

Eye-tracking features, especially the velocity of overlapping saccades and free viewing task completion time, hold potential as non-invasive biomarkers for the identification of DMF. The integration of these parameters into the XGBoost machine learning model significantly improved the accuracy of DMF diagnosis, offering a promising approach for enhancing clinical decision-making in psychiatric settings.

## 1 Introduction

Depression ranks among the most prevalent mental disorders, characterized by high incidence, recurrence, and disability rates, which impose a significant burden on society, families, and individuals (1). Approximately 11.6% of patients with depression exhibit mixed features (2), defined by the presence of typical depressive symptoms alongside some manic or hypomanic characteristics, without meeting the diagnostic criteria for mania or hypomania (3, 4). Mixed features complicate the diagnosis and treatment of depression, as conventional single antidepressant therapies may be insufficient to address the complexities of mixed presentations and potentially exacerbate manic symptoms (5, 6). Furthermore, the presence of mixed features not only increases the risk of suicide among patients (2), but also contribute to treatment resistance and a poor prognosis (7).

Diagnosing depression with mixed features (DMF) presents significant challenges in clinical practice due to the diverse and complex nature of its symptoms, which can mimic those of mania or hypomania (5). Clinicians must remain highly vigilant and conduct comprehensive assessments to accurately differentiate between the various manifestations of depression and bipolar disorder. In the absence of specific biomarkers, the diagnosis of DMF relies heavily on patients’ subjective reports and clinical observations, increasing the risk of both misdiagnosis and missed diagnosis (2). Consequently, the development of novel diagnostic strategies and the improvement of accuracy in identifying mixed features have become key research priorities in the field of mental health.

Eye-tracking technology, a non-invasive physiological measurement technique, has increasingly gained prominence in recent years within the fields of neurology, psychiatry and psychology research. This technology offers direct insights into cognitive processes, attention allocation, and emotional states by monitoring parameters such as gaze point, eye movement velocity, and eye movement trajectory (8). In the field of mental health, eye-tracking technology has been employed to investigate a variety of mental illnesses (9–11), due to its potential to elucidate the neurobiological mechanisms underlying these conditions.

Compared with healthy individuals, patients with depression exhibit distinct eye-tracking features, including a preference for negative emotional stimuli, a reduced fixation duration and number on positive stimuli, and an increased fixation duration and number on negative stimuli (12, 13). These features may serve as non-invasive biomarkers for diagnosing depression. Combining eye movement tracking with facial movement tracking can achieve up to 80% accuracy in predicting the worsening of depressive symptoms (14). Furthermore, distinct eye movement differences between mania and depression have been identified. Adolescents with depression exhibit a reduced response during the initial cognitive processing of emotional stimuli, while adolescents with mania do not significantly differ from healthy individuals (15). Eye-tracking features also differ among patients with bipolar disorder presenting different clinical manifestations. Patients with psychotic bipolar disorder exhibit more pronounced smooth pursuit dysfunction compared to those with non-psychotic bipolar disorder, which can be used to distinguish between the two (16).

While eye-tracking technology has been utilized to differentiate between patients with depression and healthy controls, as well as between individuals with bipolar disorder and those with unipolar depression (17), no studies to date have investigated the potential of eye-tracking technology in identifying MDF in patients with depression. Given the significant overlap between mixed features and manic symptoms, we hypothesize that patients with DMF will exhibit distinct eye-tracking features compared to those with pure depression. This study aims to collect and analyze eye-tracking features in patients with DMF and those without mixed features. Additionally, this study will develop a machine learning prediction model incorporating demographic data, clinical scales, and eye-tracking features to assess the potential of eye movement metrics in identifying mixed features. We anticipate that this research will provide a novel perspective for accurately identifying DMF.

## 2 Methods

### 2.1 Participants

**Figure 1:**
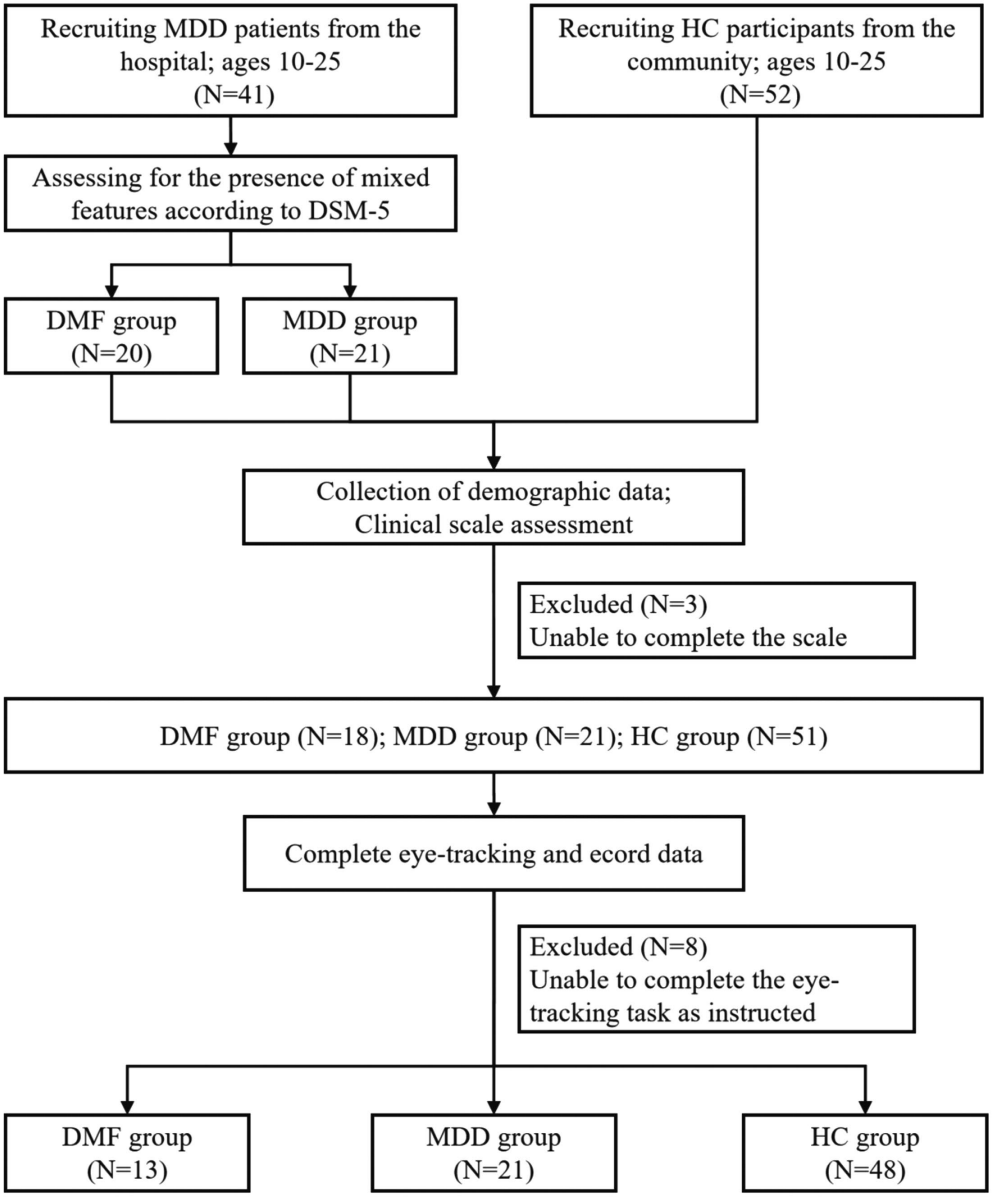
Flow chart of the study. MDD, Major depressive disorder; DMF, Depression with mixed features; HC, Healthy controls.

Our study included a total of 93 participants, consisting of 41 patients with Major Depressive Disorder (MDD) and 52 healthy controls (HC). The MDD patients underwent clinical assessments conducted by two experienced clinical psychiatrists (each holding a doctoral degree and possessing over 5 years of experience in psychiatry) and were subsequently categorized into the DMF group (*N* = 20) and the non-DMF group (labeled as MDD, *N* = 21) based on the diagnostic criteria of DSM-5. The study flowchart is presented in Figure 1.

The patient group was recruited from psychiatric inpatients at Guangdong Provincial People’s Hospital in Guangzhou, China. The inclusion criteria were as follows: (a) meeting the diagnostic criteria for MDD as specified in the DSM-5; (b) no prior physical therapy, including modified electroconvulsive therapy or repetitive transcranial magnetic stimulation; (c) aged 10–25 years; (d) of Han ethnicity; (e) right-handed; (f) able to cooperate with the study; and (g) capable of understanding the study’s content with family assistance, willing to participate, and able to provide informed consent. The exclusion criteria included: (a) comorbidity with other mental or neurological disorders; (b) MDD in clinical remission or resolved; and (c) severe physical illness.

Healthy participants were recruited via community advertisements in Guangzhou, China. The inclusion criteria for healthy participants were: (a) no mental illness as per the DSM-5; (b) no family history of mental disorders; with the remaining criteria matching those of the patient group from (c) to (g). The exclusion criteria for healthy participants were: (a) neurological diseases; and (b) severe physical illnesses.

It is important to highlight that DMF is most prevalent in children and adolescents (7), whereas the peak onset of depression typically occurs later in life. To ensure comparability of the results, we limited the age range of all study participants to 10–25 years.

### 2.2 Demographic and clinical scale assessment

This study collected demographic information, including gender, age, educational attainment, and history of leave of absence, from all participants. Each participant was interviewed by two experienced clinical psychiatrists, both holding doctorate degrees and possessing over 5 years of psychiatric experience. Interviews and standardized scales were utilized to evaluate suicide history, depression severity, mania levels, and the severity of psychotic symptoms.

The Montgomery-Asberg Depression Rating Scale (MADRS) is utilized to assess the severity of depressive symptoms (18). The Young Mania Rating Scale (YMRS) is employed to evaluate the level of mania (19). The Brief Psychiatric Rating Scale (BPRS) is utilized to assess the severity of psychotic symptoms, comprising five subscales: anxiety/depression, lack of energy, cognitive disturbance, activation, and hostility/suspiciousness (20). The Chinese version of the BPRS includes two additional items: impaired insight and work incapacity (21).

### 2.3 Eye-tracking

The eye-tracking features were recorded using a pupil center corneal reflections method that sampled at a 90-Hz rate. Eye-tracking system is name EyeKnow (Beijing CAS-Ruiyi Information Technology Co., Ltd). Prior to the experiment, a five-point calibration procedure was performed to calibrate the eye movement data with a maximum calibration error of 2° in radius. The eye-tracking features were analyzed and calculated by the eye-tracking system’s embedded data processing module. Participants received standardized and comprehensive instructions before testing to minimize potential variability. A detailed protocol was followed, outlining the sequence of tests to further reduce variability. The total time required to complete the entire task was approximately 10 min, including time for calibration, an audio-visual demonstration, and task execution.

The eye-tracking paradigms include: (1) Smooth Pursuit Tracking: A green dot moved along a sinusoidal trajectory with a horizontal/vertical/circular amplitude of 20° and a frequency of 0.2 Hz. Participants were asked to continuously track the sinusoidal movement of the target dot. Participants complete two trials. The first trial involves a single task, while the second trial requires participants to manage two tasks simultaneously, such as tracking a moving target while simultaneously remembering or identifying its characteristics. The single task assesses participants’ fundamental visuomotor processing abilities, whereas the dual-task assesses these abilities in a distracting multitasking environment. The recorded metrics for this component include latency, tracking speed, tracking acceleration, deviation count, total deviation, and mean deviation. Additionally, the single task also requires recording overshoot count, undershoot count, tracking gain, and reaction time. (2) Orienting Saccades (OS): A green target dot was displayed, and participants were instructed to rapidly shift their gaze from one point to another. This task evaluated participants’ saccadic control and the speed of their visual attention shifts. The recorded metrics included accuracy, latency, fastest completion time, average completion time, average saccade speed, and maximum saccade speed. (3) Overlapping Saccades (OLS): Participants were instructed to scan a series of overlapping targets. This task evaluated participants’ saccadic control and attention allocation abilities in processing continuous visual stimuli. The recorded metrics included accuracy, latency, fastest completion time, average completion time, average saccade speed, maximum saccade speed, overshoot count, undershoot count, multi-step saccade count, saccade gain, and maximum vertical eye movement. (4) Anti-saccades (AS): Similar to orienting saccades, participants were instructed to execute a saccade to the opposite position of the target dot as quickly as possible. This task evaluated participants’ inhibitory control and proactive attention shifting abilities. The recorded metrics included accuracy, latency, fastest completion time, average completion time, uncorrected error rate, average saccade speed, maximum saccade speed, and maximum vertical eye movement. (5) Free Viewing (FV): The task allowed participants to freely observe a scene without specific instructions. This task evaluated participants’ natural visual exploration behavior and attention allocation. The recorded metrics included saccade count, fixation count, and task average completion time. (6) Go/No Go Task; Participants were required to respond to a series of stimuli, where some stimuli (Go) required a response, while others (No Go) required inhibiting a response. This task evaluated participants’ selective attention and inhibitory control abilities. The recorded metrics included accuracy and latency. For data collection methods across different paradigms, refer to relevant research papers (12, 22, 23).

### 2.4 Machine learning (ML)

We constructed a dataset using baseline demographic characteristics such as gender, age, educational attainment, and school absenteeism, along with clinical questionnaires (BPRS, MADRS, YMRS) and eye movement data (OS, OLS, AS, FV). These features were used to train a model to predict whether each patient with depression exhibits mixed features. The data were normalized prior to analysis. The dataset was then randomly divided into a training set and a test set in a 7:3 ratio.

The machine learning algorithm employing extreme gradient boosting (XGBoost), implemented using the XGBoost library in Python 3.7, was used to develop predictive models that effectively perform classification tasks. To investigate the correlation between eye movement parameters and mixed features, two distinct feature sets were developed. XGBoost-1 was trained using demographic characteristics (gender, age, educational attainment, and school absenteeism) and clinical questionnaires (BPRS, MADRS, YMRS), without including any eye movement parameters. In contrast, XGBoost-2 includes the aforementioned characteristics and eye movement parameters (OS, OLS, AS, FV). To optimize the performance of the XGBoost model, we systematically tuned the key hyperparameters. Supplementary Table 1 shows the ranges of the hyperparameters that were tuned and the final optimal parameter values that were determined. Supplementary Figure 1 illustrates the changes in cross—validation AUC scores during the Bayesian optimization process. The model’s predictive discrimination ability was evaluated using accuracy, precision, recall, F1 score, and area under the curve (AUC), calculated using R-Studio 4.2.2. Model performance was compared using AUC, decision curve analysis (DCA), and net reclassification improvement (NRI). The AUC of the two models was compared using the DeLong test in MedCalc 22.021. Additionally, the local explanation technique SHAP (Shapley Additive Explanations) was used to identify which features significantly influence the model’s output and to elucidate the model’s decision-making process.

### 2.5 Statistical analysis

Descriptive analyses were conducted on the demographic data and clinical symptom assessment data from the three subject groups in this study, including demographic characteristics, clinical questionnaires, and eye movement data. The analysis of variance (ANOVA) F test and the Pearson chi-square test were used to compare continuous and categorical data among the three groups: DMF, MDD, and HC. Groups that exhibited statistically significant differences after the analysis of variance underwent further *post hoc* comparisons. Spearman correlation analysis was used to assess the relationship between disease group scale scores and eye movement parameters. The significance level was set at *p* < 0.05. All statistical analyses were performed using SPSS version 26.0.

## 3 Results

### 3.1 Demographic and clinical characteristics

This study included 93 participants, comprising 20 individuals with DMF, 21 with MDD, and 52 healthy controls (HC). Table 1 presents the demographic and clinical characteristics across the three groups. A higher proportion of women was observed in both the DMF and MDD groups, but the statistical difference between these two groups was not significant (Female: DMF, 85%; MDD, 76.2%; *χ*^2^ = 0.506, *p* = 0.477; HC, 50%). There were no dropouts in the HC group, while a proportion of participants in both the DMF and MDD groups did drop out of school. The difference in dropout rates between these two groups was also not statistically significant (School Dropout: DMF, 35%; MDD, 19%; *χ*^2^ = 1.328, *p* = 0.249). Both the DMF and MDD groups exhibited suicidal ideation or behavior, with no significant statistical difference between them (*χ*^2^ = 1.205, *p* = 0.272). In terms of clinical characteristics, with the exception of the Young Mania Rating Scale (YMRS) results, no statistically significant differences were found between the DMF group and the MDD group (all *p* > 0.05). The Impaired Insight score was significantly higher in the DMF group than in the HC group (*t* = −2.878, *p* = 0.005), although the difference from the MDD group was not statistically significant. Additionally, the difference in Impaired Insight scores between the HC group and the MDD group was not statistically significant. Regarding YMRS scores, the DMF group exhibited significantly higher scores than both the MDD group and the HC group (DMF vs. MDD: *t* = 3.041, *p* = 0.006; DMF vs. HC: *t* = −3.652, *p* = 0.002), while the statistical difference between the MDD and HC groups was not significant (MDD vs. HC: *t* = −1.594, *p* = 0.126).

**Table 1:** Demographic and clinical features.

Items	DMF (*N* = 20) Mean (sd)/*N* (%)	MDD (*N* = 21) Mean (sd)/*N* (%)	HC (*N* = 52) Mean (sd)/*N* (%)	Significant direction
Age	18.05 (3.35)	17.52 (3.49)	17.79 (7.93)	
Gender				Female: DMF, MDD > HC
Male	3 (15%)	5 (23.8%)	26 (50%)	
Female	17 (85%)	16 (76.2%)	26 (50%)	
Educational attainment				
<9	7 (35%)	6 (28.6%)	5 (9.6%)	
9–12	8 (40%)	10 (47.6%)	32 (61.5%)	
>12	5 (25%)	5 (23.8%)	15 (28.8%)	
School dropout				HC without dropout
No	13 (65%)	17 (81%)	52 (100%)	
Yes	7 (35%)	4 (19%)	/	
Suicidal ideation and behavior				HC without suicidal ideation and behavior
No	/	/	52 (100%)	
Suicidal ideation only	8 (40%)	12 (57.1%)	/	
Suicide behavior	12 (60%)	9 (42.9%)	/	
BPRS total score	31.11 (7.59)	30.57 (8.03)	19.02 (1.42)	DMF, MDD > HC
Anxiety/depression	2.51 (0.93)	2.72 (0.75)	1.17 (0.29)	DMF, MDD > HC
Lack of energy	1.45 (0.60)	1.59 (0.79)	1.04 (0.10)	DMF, MDD > HC
Cognitive disturbance	1.52 (0.67)	1.25 (0.48)	1.02 (0.07)	DMF, MDD > HC
Activation	1.71 (0.57)	1.51 (0.41)	1.04 (0.10)	DMF, MDD > HC
Hostility/suspiciousness	1.44 (0.53)	1.31 (0.48)	1.02 (0.07)	DMF, MDD > HC
BPRS additional questions				
Impaired insight	1.39 (0.98)	1.1 (0.44)	1 (0)	DMF > HC
Work incapacity	1.78 (1.40)	2 (1.41)	1 (0)	DMF, MDD > HC
MADRS	35.78 (12.55)	37.86 (10.34)	11.43 (2.98)	DMF, MDD > HC
YMRS	8.39 (9.37)	1.38 (3.03)	0.31 (0.81)	DMF > MDD、HC

DMF, Depression with mixed features; MDD, Major depressive disorder; HC, Healthy controls; BPRS, Brief Psychiatric Rating Scale; MADRS, Montgomery-Asberg Depression Rating Scale; YMRS, Young Mania Rating Scale.

### 3.2 Eye-tracking features

Table 2 presents the eye-tracking features for each group. The metrics for smooth pursuit tracking (both dual task and single task), eye fixations, and responsive search score did not show statistically significant differences among the three groups. However, several indicators related to OS, OLS, AS, FV, and the Go/NoGo Task showed statistically significant differences among the groups (*p* < 0.05). The specific indicators are as follows: OS-fastest completion time (*F* = 3.385, *p* = 0.039), OS-maximum saccade speed (*F* = 3.577, *p* = 0.033), OLS-fastest completion time (*F* = 3.128, *p* = 0.049), OLS-average saccade speed (*F* = 4.233, *p* = 0.01), OLS-maximum saccade speed (*F* = 3.403, *p* = 0.038), OLS-undershoot count (*F* = 3.264, *p* = 0.043), OLS-saccade gain (*F* = 4.245, *p* = 0.018), AS-average completion Time (*F* = 3.181, *p* = 0.047), FV-saccade count (*F* = 8.028, *p* = 0.001), FV-fixation count (*F* = 7.583, *p* = 0.001), FV-task average completion time (*F* = 3.378, *p* = 0.039), Go/NoGo-accuracy (*F* = 3.922, *p* = 0.024).

**Table 2:** Oculometric parameters.

Items	DMF (*N* = 13) Mean (sd)	MDD (*N* = 21) Mean (sd)	HC (*N* = 48) Mean (sd)	ANOVA *p*	DMF vs. MDD	DMF vs. HC	MDD vs. HC
Smooth pursuit tracking (dual task)							
Latency (ms)	754.74 (537.94)	1,307.04 (3,207.74)	707.18 (838.98)	0.415			
Tracking speed (°/s)	20.25 (10.88)	40.34 (47.2)	26.43 (18.5)	0.084			
Tracking acceleration (°/s^2^)	38.58 (38.78)	268.44 (697.13)	167.66 (708.64)	0.605			
Deviation count (times)	25.46 (18.66)	22.57 (16.94)	20.67 (18.79)	0.693			
Total deviation (>4°, °)	140.25 (104.96)	140.41 (111.43)	118.36 (107.42)	0.664			
Mean deviation (>4°, °)	5.46 (0.8)	5.84 (1.93)	5.38 (1.58)	0.549			
Smooth pursuit tracking (single task)							
Latency (ms)	641.31 (250.7)	580.13 (263.14)	577.14 (339.01)	0.795			
Tracking speed (°/s)	20.81 (5.32)	32.07 (36.75)	40.09 (72.12)	0.558			
Tracking acceleration (°/s^2^)	41.05 (25.86)	889.93 (3,822.71)	1,923.84 (9,479.09)	0.691			
Deviation count (times)	36.31 (19.76)	32.67 (25.57)	24.44 (23.72)	0.182			
Total deviation (>4°, °)	192.85 (107.93)	181.74 (140.73)	132.83 (124.34)	0.174			
Mean deviation (>4°, °)	5.37 (0.67)	5.63 (0.69)	5.61 (1.27)	0.758			
Overshoot count (times)	8.77 (8.04)	7.86 (7.7)	6.31 (4.83)	0.37			
Undershoot count (times)	16.15 (13.24)	10.52 (9.11)	8.98 (11.26)	0.125			
Tracking gain (−)	80.24 (41.47)	66.66 (28.83)	59.76 (48.48)	0.315			
Reaction time (ms)	514.79 (176.77)	463.59 (200.63)	434.03 (146.18)	0.292			
Orienting saccades							
Accuracy (%)	100 (0)	97.93 (5.24)	99.7 (2.06)	0.065			
Latency (ms)	276.31 (56.65)	266.06 (52.25)	260.2 (30.75)	0.458			
Fastest completion time (ms)	270.76 (74.59)	240.48 (54.22)	233.68 (28.71)	0.039	0.191	0.033	1
Average completion time (ms)	343.34 (83.75)	309.41 (107.48)	292.46 (37.5)	0.067			
Average saccade speed (°/s)	209.21 (119.47)	281.98 (131.21)	282.01 (68.46)	0.050			
Maximum saccade speed (°/s)	420.32 (212.07)	565.66 (192.73)	500.5 (113.98)	0.033	0.028	0.305	0.335
Overlapping saccades							
Accuracy (%)	99.3 (2.52)	98.61 (4.54)	99.83 (1.2)	0.216			
Latency (ms)	333.4 (65.35)	317.1 (103.07)	312.2 (64.86)	0.676			
Fastest completion time (ms)	292.21 (86.58)	233.59 (76.66)	248.91 (57.17)	0.049	0.049	0.132	1
Average completion time (ms)	408.42 (71.51)	359.12 (117.33)	351.12 (71.87)	0.106			
Average saccade speed (°/s)	218.89 (94.17)	292.36 (105.08)	291.64 (67.84)	0.018	0.042	0.019	1
Maximum saccade speed (°/s)	506.47 (247.15)	648.42 (183.38)	559.89 (125.76)	0.038	0.049	0.910	0.131
Overshoot count (times)	0.46 (0.78)	1.1 (1.26)	0.58 (1.11)	0.155			
Undershoot count (times)	2.15 (2.3)	1.57 (1.81)	0.9 (1.45)	0.043	1	0.060	0.396
Multi-step saccade count (times)	0 (0)	0.05 (0.22)	0 (0)	0.236			
Saccade gain	0.88 (0.18)	0.94 (0.12)	0.98 (0.09)	0.018	0.300	0.016	0.712
Maximum vertical eye movement (Up, °)	18.58 (3.91)	19.06 (3.25)	18.46 (3.58)	0.813			
Maximum vertical eye movement (Down, °)	20.85 (3.27)	19.44 (4.04)	20.11 (5.2)	0.691			
Maximum horizontal eye movement (Left, °)	18.04 (3.19)	19.86 (5.83)	20.27 (5.06)	0.374			
Maximum horizontal eye movement (Right, °)	18.89 (4.32)	21.22 (7.3)	20.23 (5.08)	0.503			
Anti-saccades							
Accuracy (%)	55.32 (37.65)	65.94 (20.97)	64.65 (26.59)	0.496			
Latency (ms)	320.69 (74.67)	337.98 (57.85)	321.82 (52.15)	0.532			
Fastest completion time (ms)	293.41 (134.54)	302.96 (129.67)	260.14 (47.29)	0.16			
Average completion time (ms)	445.81 (145.59)	420.92 (116.64)	376.99 (71.65)	0.047	1	0.085	0.277
Uncorrected error rate	21.08 (36.48)	10.78 (12.85)	9.61 (17.43)	0.206			
Average saccade speed (°/s)	205.96 (117.81)	249.48 (109.53)	254.54 (77.2)	0.249			
Maximum saccade speed (°/s)	508.91 (243.47)	621.85 (243.65)	556.53 (181.33)	0.282			
Maximum vertical eye movement (Up, °)	20.43 (11.3)	25.16 (7.28)	25.84 (7.92)	0.122			
Maximum vertical eye movement (Down, °)	23.21 (9.59)	22.84 (7.57)	25.87 (6.54)	0.219			
Maximum horizontal eye movement (Left, °)	29.13 (11.79)	32.27 (7.06)	32.58 (8.35)	0.441			
Maximum horizontal eye movement (Right, °)	30.17 (13.48)	30.56 (7.29)	33.57 (7.45)	0.265			
Free viewing							
Saccade count (times)	34 (7.79)	38.95 (4.73)	32.56 (6.14)	0.001	0.073	1	<0.001
Fixation count (number)	35.15 (8.07)	39.71 (4.73)	33.42 (6.17)	0.001	0.119	1	0.001
Task average Completion time (s)	0.93 (0.43)	1.11 (0.28)	1.22 (0.37)	0.039	0.527	0.039	0.713
Go/NoGo task							
Accuracy (%)	58.46 (29.96)	49.05 (22.34)	67.08 (24.49)	0.024	0.861	0.814	0.021
Latency (ms)	352.3 (121.29)	330.35 (132.97)	363.82 (116.13)	0.576			

DMF, Depression with mixed features; MDD, Major depressive disorder; HC, Healthy controls.

**Figure 2:**
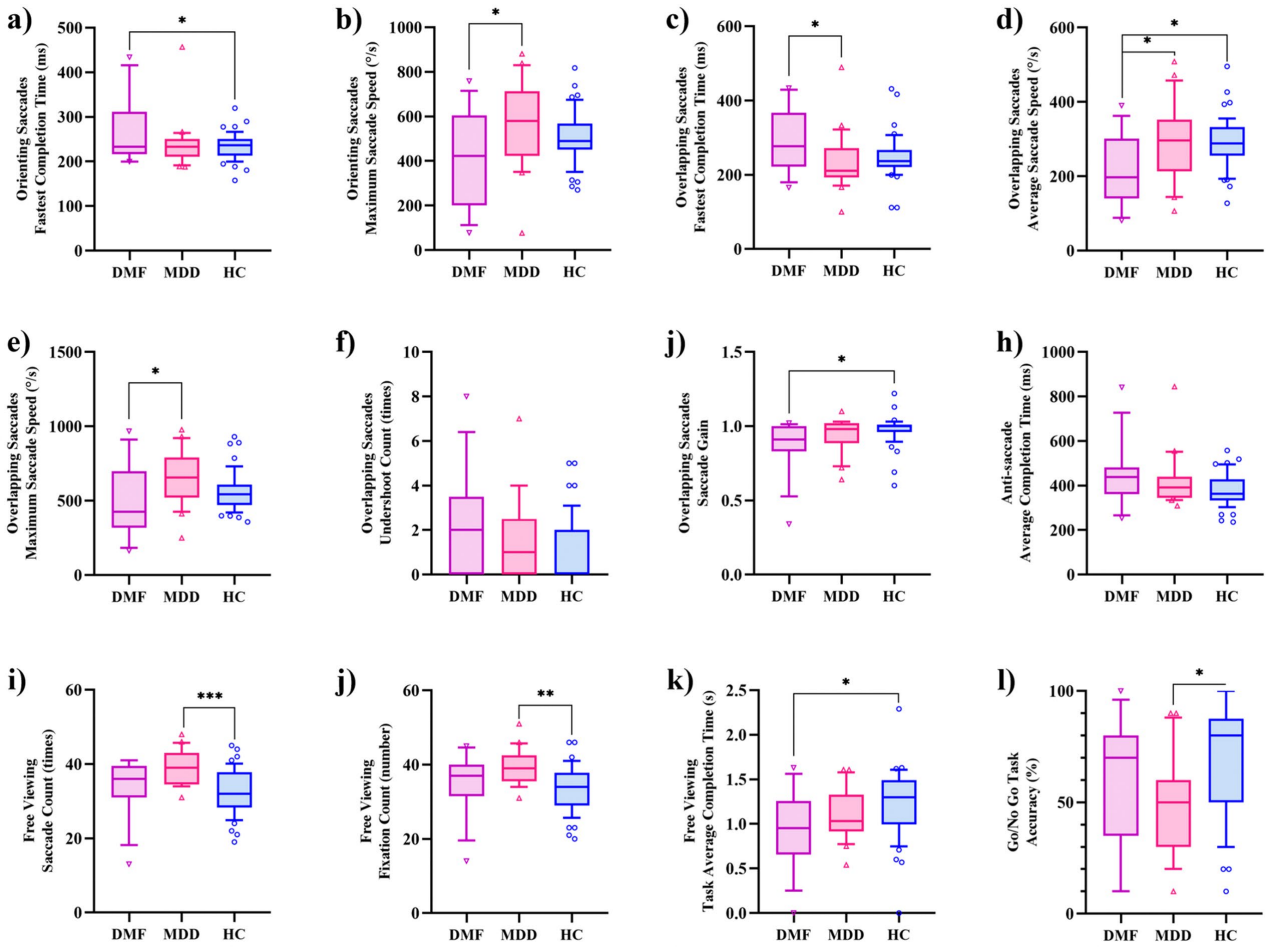
Inter-group differences in eye-tracking features. **(a)** Orienting saccades: fastest completion time; **(b)** Orienting saccades: maximum saccade speed; **(c)** Overlapping saccades: fastest completion time; **(d)** Overlapping saccades: average saccade speed; **(e)** Overlapping saccades: maximum saccade speed; **(f)** Overlapping saccades: undershoot count; **(g)** Overlapping saccades: saccade sain; **(h)** Anti-saccades: average completion time; **(i)** Free viewing: saccade count; **(j)** Free viewing: fixation count; **(k)** Free viewing: task average completion time; **(l)** Go/NoGo task: accuracy.

Figure 2 displays the results of *post hoc* comparisons among the three groups. The OS-maximum saccade speed, OLS-average saccade speed, and OLS-maximum saccade speed were slower in the DMF than in the MDD, while the OLS-fastest completion time was longer in the DMF than in the MDD. No statistically significant differences were observed for the remaining indicators between the DMF and MDD groups. The indicators that showed differences between the DMF and HC include OS-fastest completion time, OLS-average saccade speed, OLS-saccade gain, and FV-task average completion time. In contrast, the indicators that showed differences between the MDD and HC are FV-saccade count, FV-fixation count, and Go/NoGo accuracy.

### 3.3 Correlation analysis between eye-tracking features and clinical scales

**Figure 3:**
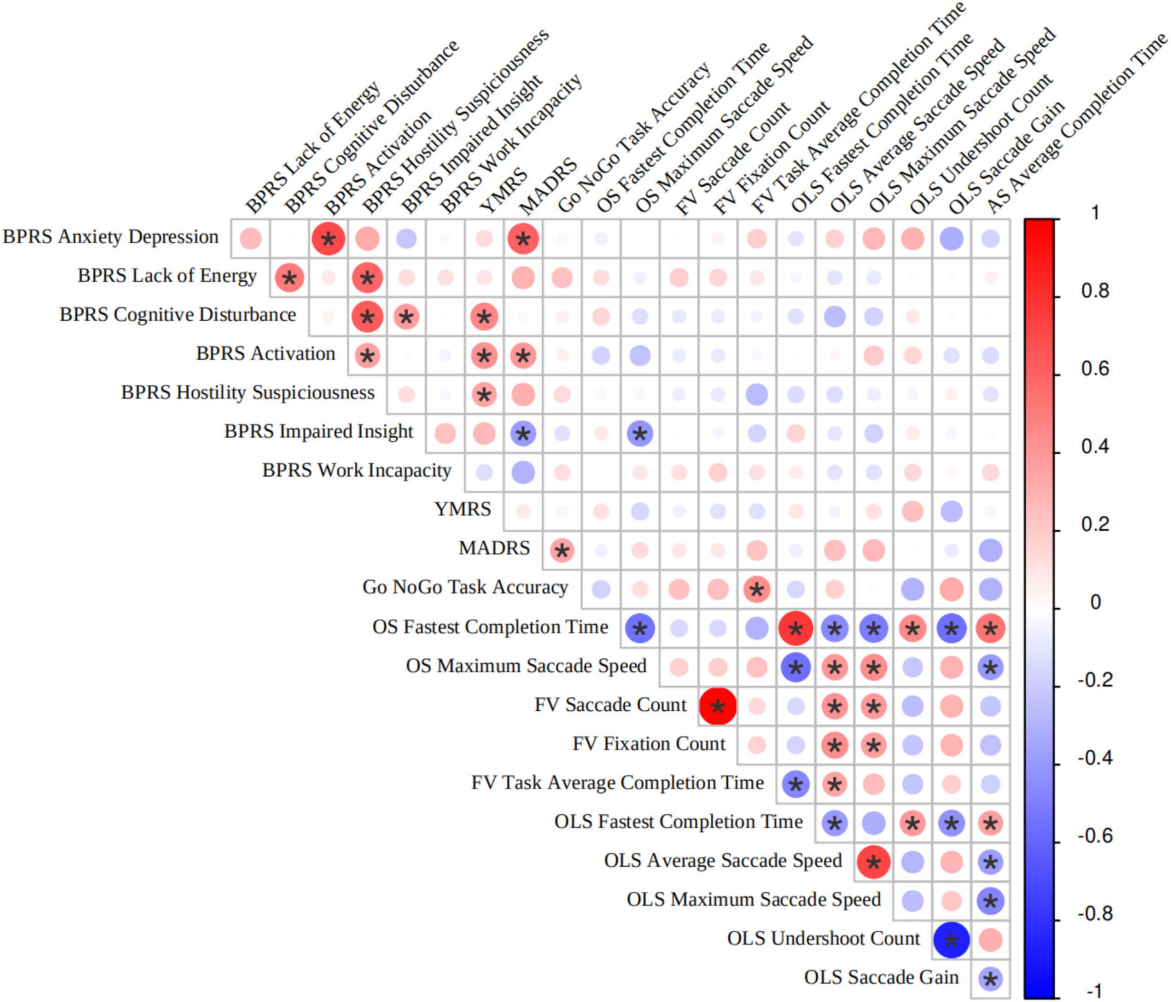
Heatmap of correlations between scale results and eye-tracking features. OLS, overlapping saccades; OS, Orienting saccades; FV, Free viewing; AS, Anti-saccades; BPRS, Brief Psychiatric Rating Scale; MADRS, Montgomery-Asberg Depression Rating Scale; YMRS, Young Mania Rating Scale.

Figure 3 presents the correlation analysis between eye-tracking features and clinical scales in the DMF and MDD. Notably, only the OS-maximum saccade speed showed a negative correlation with BPRS-impaired insight (*p* < 0.05), while no significant correlations were observed between other eye-tracking features and clinical scales. These findings suggest that eye-tracking features represent a distinct set of characteristic data for patients with depression, independent of clinical scale scores.

### 3.4 Predictive value of eye-tracking features for DMF

**Figure 4:**
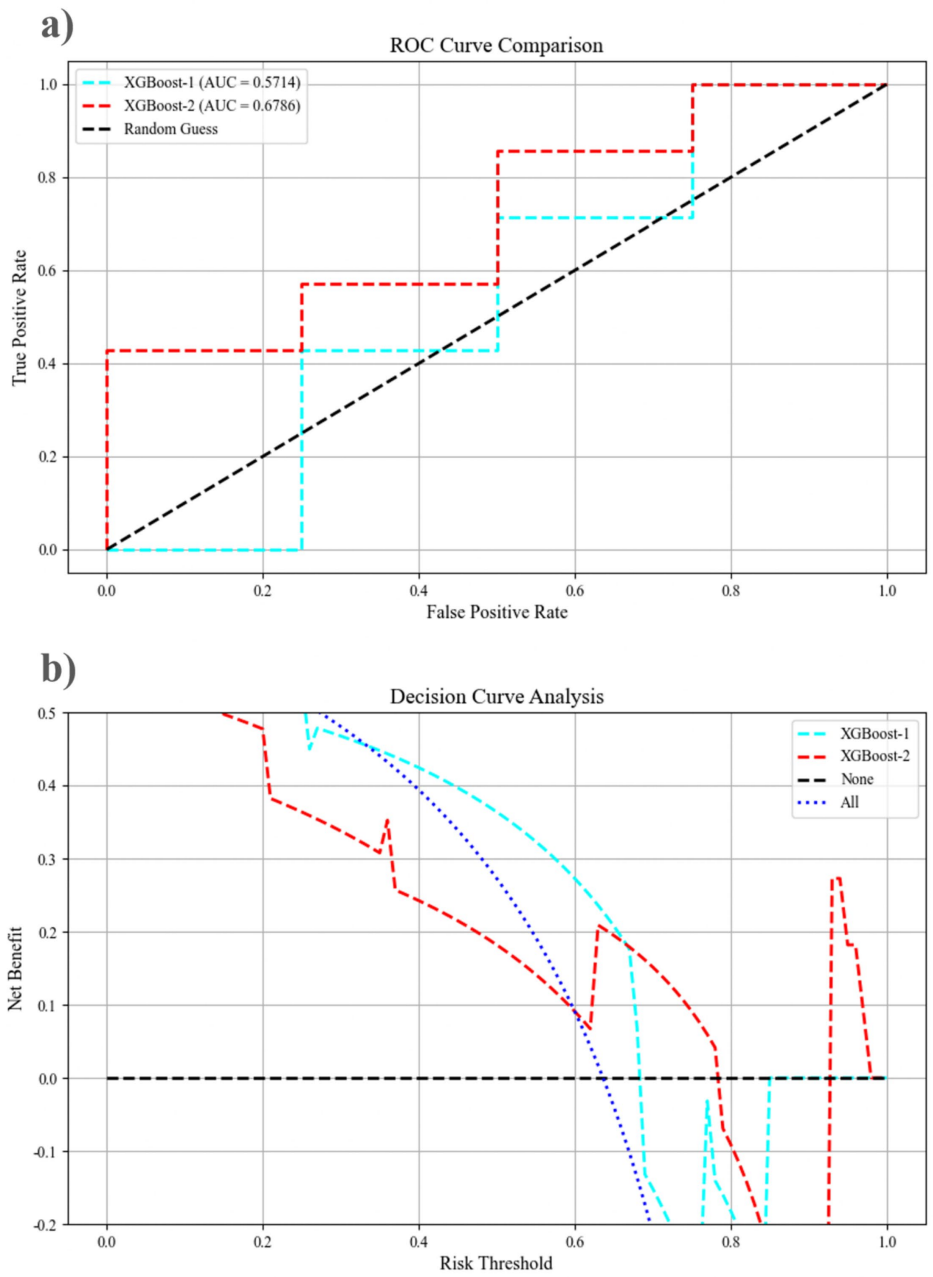
The performance of the mixed features pre-prediction model, as assessed by the receiver operating curve and decision curve analysis, for XGBoost-1 and XGBoost-2 on the testing dataset. **(a)** The ROC curve comparison; **(b)** Decision curve analysis. AUC, area under the curve; ROC, receiver operating characteristic.

The dataset was randomly divided into two groups: a training set comprising 70% of the 34 patients and a test set consisting of the remaining 30%. XGBoost-1 was trained using demographic and clinical features, achieving an area under the curve (AUC) of 0.571 (95% confidence interval [CI]: 0.143, 0.964) in the test set. When additional eye movement parameters were incorporated, including OS-fastest completion time, OS-maximum saccade speed, OLS-fastest completion time, OLS-average saccade speed, OLS-maximum saccade speed, OLS-undershoot count, OLS-saccade gain, AS-average completion time, FV-saccade count, FV-fixation count, FV-task average completion time, and Go/NoGo-accuracy, the AUC of XGBoost-2 improved to 0.679 (95% CI: 0.321, 0.964) (Figure 4). The DeLong test indicates that the AUC of the XGBoost algorithm improved by 0.108 (*p* = 0.039), reflecting an 18% enhancement in performance. Additionally, the metrics of accuracy, precision, recall, and F1 score increased by 33.2, 12.4, 50.1, and 18.9%, respectively (Table 3). DCA shows that when the risk threshold is set between 0.69 and 0.78, XGBoost-2 exhibits superior diagnostic efficiency and net benefit compared to XGBoost-1.

**Table 3:** Prediction results of XGBoost.

	Accuracy	Precision	Recall	F1 score	AUC
XGBoost-1	0.546 (0.273, 0.818)	0.667 (0.333, 1.000)	0.571 (0.143, 0.861)	0.615 (0.200, 0.857)	0.571 (0.143, 0.964)
XGBoost-2	0.727 (0.455, 0.909)	0.750 (0.571, 1.000)	0.857 (0.571, 1.000)	0.800 (0.571, 0.933)	0.679 (0.321, 0.964)

All are 95% confidence intervals (CI). AUC, Area under the curve.

### 3.5 Eye-tracking features prediction the optimal feature set of DMF

**Figure 5:**
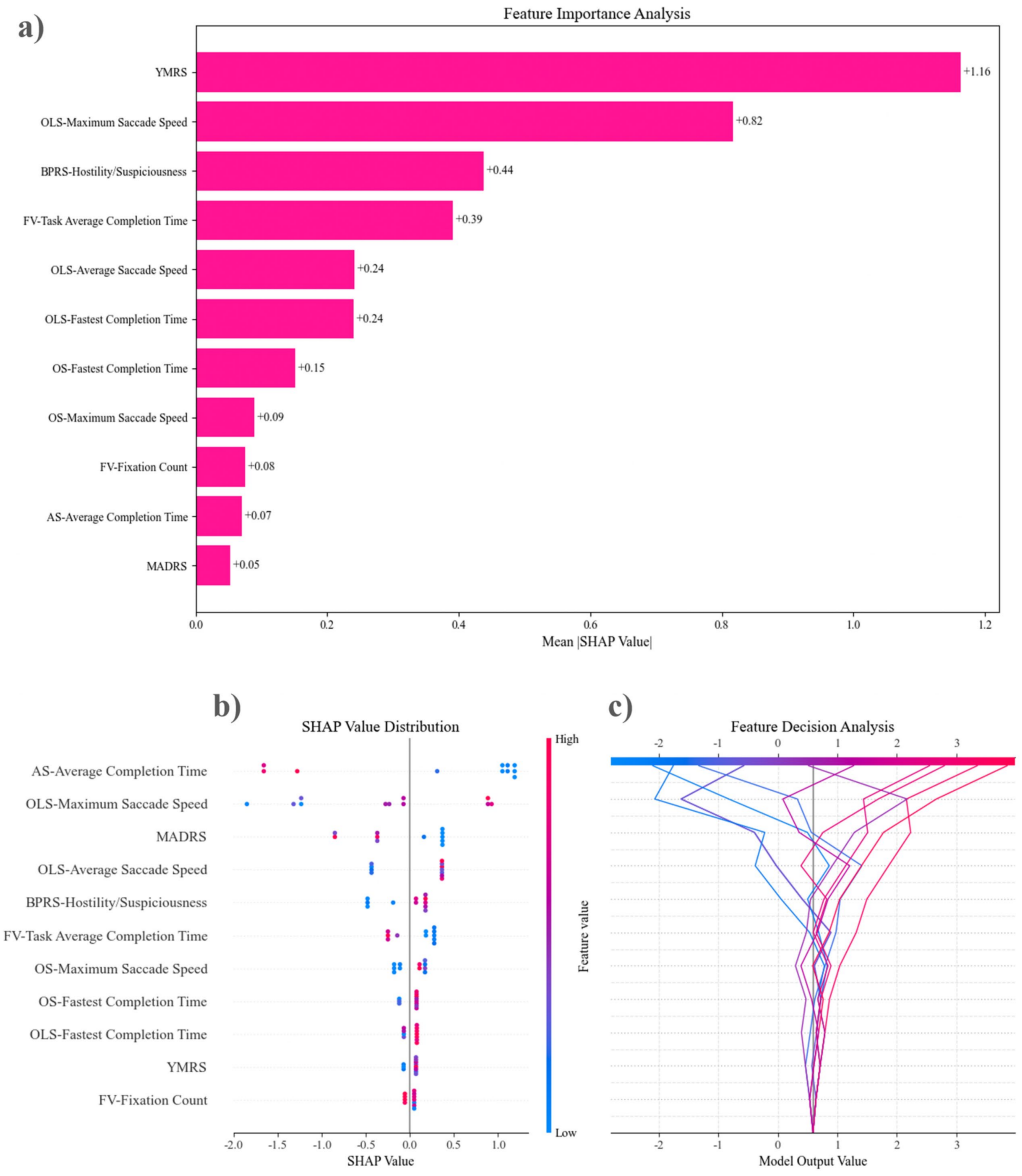
The feature sets of the top-performing model were evaluated using SHAP analysis. **(a)** Features are ordered from the most influential to the least, based on their contribution to the predictive accuracy of XGBoost-2 in forecasting mixed features. **(b)** Feature Decision Analysis illustrates how the model utilizes these features to make decisions. The lines in the figure depict the trajectory of changes in model output values as feature values vary. It can be observed that changes in AS—Average Completion Time lead to significant fluctuations in model output values (within a range of approximately −2 to 2), while features at the bottom, such as FV—Fixation Count, have relatively smaller impacts on the model output. **(c)** The SHAP Value Distribution shows the contribution distribution of each feature to the model’s prediction results. The influence of AS—Average Completion Time is the most significant. When its value is high (red points), it tends to have a positive contribution to the prediction results, while a low value (blue points) has a negative contribution. OLS—Maximum Saccade Speed and MADRS also show strong predictive power. OLS, overlapping saccades; OS, Orienting saccades; FV, Free viewing; AS, Anti-saccades; BPRS, Brief Psychiatric Rating Scale; MADRS, Montgomery-Asberg Depression Rating Scale; YMRS, Young Mania Rating Scale.

Figure 5 illustrates the optimal feature set for the XGBoost-2 model. The YMRS emerges as the most significant predictor of XGBoost-2 classification, followed closely by OLS-maximum saccade speed and BPRS-hostility/suspiciousness. Among the eye-tracking features, the top three predictive factors are OLS-maximum saccade speed, FV-task average completion time, and OLS-average saccade speed. The SHAP analysis indicates that a longer AS-average completion time, a slower OLS-maximum saccade speed, and lower MADRS scores are closely associated with the identification of mixed features. This evidence supports the classification of these features as DMF by the XGBoost-2 model. By integrating results from eye-tracking features (OLS, AS, FV) and clinical scales (YMRS, MADRS), the predictive capacity of the XGBoost model for DMF is enhanced.

## 4 Discussion

This study investigated the relationship between eye-tracking features and depression, aiming to develop a prediction model for mixed features using machine learning methods. A key finding was the identification of significant differences in eye-tracking features among DMF, MDD, and HC. Notably, in certain indicators of OS and OLS, the DMF exhibited slower maximum and average saccade speeds, as well as longer fastest completion times compared to the MDD. Furthermore, the DMF demonstrated significantly more saccades and fixations during the FV than the HC, suggesting that DMF may experience specific cognitive impairments related to visual attention and processing speed. Another significant finding was that incorporating eye-tracking features into the machine learning prediction model markedly enhanced its ability to predict DMF. This indicates that eye-tracking features provide valuable additional information for identifying DMF, underscoring their potential role in diagnosing mental health disorders.

Patients with depression exhibit abnormalities in eye-tracking features, potentially linked to the neural mechanisms underlying emotion regulation and cognitive control (13). We found that eye-tracking features are also correlated with DMF. Notably, the reduced performance of DMF patients in the maximum saccade speed of OS and the average saccade speed of OLS may indicate distinct neurocognitive substrates compared to patients with non-mixed depression. These abnormalities may reflect a reduced ability to process emotional stimuli, which is consistent with the attentional bias and emotional regulation deficits often observed in patients with depression (5). A decreased ability to process emotional stimuli often indicates a poorer prognosis (24). Moreover, the observed slowing of eye-tracking features may be associated with functional abnormalities in the prefrontal cortex and limbic system, which are crucial for regulating attention and emotional responses (25, 26). Some studies suggest that patients with bipolar disorder exhibit varying attention preferences for emotion-related images at different clinical stages, potentially linked to alterations in eye movement parameters (27). Our findings support this notion and specifically focus on DMF, a subgroup believed to share certain characteristics with patients diagnosed with bipolar disorder, yet have received comparatively less attention in previous studies (6).

Eye-tracking technology has garnered significant attention for its applications in mental disorder-related conditions, due to its non-invasive nature and its ability to directly measure visual attention allocation. This technology can reveal patients’ attention biases when processing emotional stimuli, thereby aiding in understanding the pathological mechanisms underlying depression (28). Researchers have utilized eye tracking within the emotion recognition paradigm to explore the connections between MDD, childhood trauma, and emotional processing (29). In terms of disease diagnosis, the parameters derived from eye tracking provide an objective tool for diagnosis, thereby reducing reliance on patients’ subjective reports (14). Our study also found that there were certain correlations among the BPRS, YMRS, and MADRS scores in MDD patients, while there were almost no correlations between eye-tracking features and the above—mentioned scale results. This confirms that eye-tracking features are a set of independent parameters that can bypass the ambiguity of scales and be included in the objective diagnostic markers for MDD. Moreover, the application of eye-tracking technology can go beyond diagnosis; it can also be used to monitor disease progression and evaluate treatment effectiveness. By identifying patients’ responses to treatment over an extended period, clinicians can adjust treatment plans in a timely manner for more precise interventions. Currently, some researchers are investigating the use of eye-tracking technology for long-term assessment of the severity of depression in order to better adjust treatment strategies (30).

Identifying mixed features in patients with depression presents a significant challenge in psychiatry. Table 4 summarizes the advantages and disadvantages of the existing diagnostic schemes for MDF. Current diagnostic standards primarily rely on symptomatology, leading to a high rate of missed diagnoses and misdiagnoses. Some studies have used the YMRS to identify DMF, employing criteria such as a Depressive Symptomatology-Clinician-Rated Version score ≥ 15 and a YMRS score between 2 and 12. However, researchers have noted that existing scales are insufficient for detecting primary DMF (31, 32). In this study, the XGBoost-1 model, incorporating demographic and clinical scales (including YMRS), yielded an AUC of only 0.571, indicating that common clinical scales may lack reliability in identifying DMF, consistent with previous findings. In contrast, the XGBoost-2 model, integrating eye-tracking features, achieved an AUC of 0.679, representing an 18.9% improvement, along with enhanced accuracy and precision. SHAP analysis identified that certain indicators within OLS, AS, and FV significantly influence the prediction model. These results provide evidence that eye-tracking, an emerging auxiliary technology, can be applied in clinical settings. As an objective indicator, clinicians may consider incorporating eye-tracking features into early screening protocols for DMF to achieve more accurate diagnoses.

**Table 4:** Advantages and disadvantages of MDF diagnostic schemes.

Items	Description	Advantages	Disadvantages
Clinical mental examination	Based on DSM-5 or ICD-11	Currently the authoritative standard for diagnosing DMF	Subjective; relies on the professional level of the clinician Time window limitation exists (symptoms lasting ≥2 weeks), which may delay early identification
Psychiatric scales	YMRS, HCL-32, etc.	Quantitative assessment Can be used for large-scale screening	Still relatively subjective, with patient self-report bias
Traditional biomarkers	Metabolomics, genetic polymorphisms, inflammatory markers, etc.	Objective Can be used for early identification	High cost Lack of specificity Most biomarkers are still in the research stage, lacking clinical translation
Emerging auxiliary techniques	Eye-tracking features, machine learning models, voice feature, etc.	Objective Can integrate multimodal data	Lack of clinical evidence

DMF, Depression with mixed features; DSM-5, Diagnostic and Statistical Manual of Mental Disorders, Fifth Edition; ICD-11, Eleventh Revision of the International Statistical Classification of Diseases and Related Health Problems; YMRS, Young Mania Rating Scale; HCL-32, Hypomania Checklist-32.

Additionally, the application of ML in neurological and psychiatric disorders is becoming an increasingly important trend. By analyzing extensive clinical, psychological, and biological data, machine learning models can identify patterns and trends related to neurological and psychiatric disorders, thereby supporting the prediction, diagnosis, and treatment of these conditions. For instance, 14 types of ML algorithms are effective for early detection of cognitive impairment (33). In depression research, XGBoost and network analysis help identify depression-related factors and their relationships, and can be applied to epidemiological studies using large survey data (34, 35). By incorporating multimodal data and combining it with ML, depression diagnosis technology may further develop, particularly in improving the accuracy of identifying depression subtypes.

When using ML in research, it is crucial to be cautious about over-fitting, as it can lead to a model performing well on the training data but poorly on unseen data, thereby severely affecting the model’s generalization ability. In this study, we used the Bayesian optimization algorithm to automatically search for the optimal hyperparameter combination for the XGBoost model: limiting the maximum depth of trees (max depth: 3–10) to prevent the trees from becoming too complex, using gamma (0–1) and min child weight (1–10) parameters to control the threshold for tree node splitting to reduce model complexity, adopting a small learning rate range (0.01–0.3) to stabilize model training, and combining 5-fold cross-validation (cv. = 5) to evaluate the model’s performance on different data subsets. This optimization strategy can effectively prevent over-fitting while maintaining the model’s predictive performance.

This study does have certain limitations. Firstly, the relatively small sample size may have limited the statistical power and generalizability of the results. Secondly, participants were recruited from specific geographical locations and cultural backgrounds, which may affect the representativeness of the sample and the applicability of the findings. In visual search tasks, Saudi participants were less efficient in their search behavior than British participants (36); in fixation tasks, Czech participants focused more on the focal objects measured by fixation counts, while participants from Taiwan spent more time fixating on the background (37); hence, studies involving a more diverse population are necessary to validate our results. Additionally, the measurement of eye-tracking features could be influenced by individual differences, such as medication use or cognitive ability (38, 39), and these factors may not have been adequately controlled in this study. Lastly, while we utilized common machine learning models for data analysis, the predictive accuracy of these models may be influenced by data quality and feature selection. Model performance may vary across different datasets and clinical settings, indicating that further validation studies are essential to assess the model’s robustness.

Based on the findings and limitations of this study, we suggest several avenues for future research. Firstly, expanding the sample size and enhancing the diversity of research participants is crucial. Secondly, conducting long-term follow-up studies will help clarify changes in eye movement parameters over time and their correlation with the progression and treatment efficacy of depression. Additionally, standardized procedures for the clinical application of eye-tracking should be gradually established to reduce information bias, which relies on long-term clinical research and practice. Finally, it is important to investigate other potential biomarkers beyond eye-tracking features, such as neuroimaging markers, genetic markers, and behavioral characteristics (40–43). Developing a more comprehensive hybrid feature recognition framework by integrating multi-modal data could significantly enhance diagnostic accuracy.

## 5 Conclusion

This study underscores the potential value of eye-tracking technology in identifying DMF. Eye-tracking features may serve as non-invasive biomarkers that enhance the objectivity and accuracy of depression diagnoses. By integrating data on eye-tracking features, the application of ML models reveals significant potential for identifying DMF. This data-driven approach promises to yield more precise predictions for clinical decision-making. Future research and clinical practice should further explore and validate these findings to achieve accurate diagnosis and treatment of depression.

## Data Availability

The raw data supporting the conclusions of this article will be made available by the authors without undue reservation.

## Supplementary material

SUPPLEMENTARY FIGURE 1 The changes in cross-validation AUC scores during the Bayesian optimization process. As the number of iterations increases, the AUC scores gradually improve and stabilize after a certain iteration. The red dot in the figure indicates the best AUC score and the corresponding iteration number obtained throughout the optimization process. Through this optimization method, the model can iteratively adjust the hyperparameters to achieve optimal performance.
