# Supplementary material for: Identifying depression with mixed features: the potential value of eye-tracking features
Source: Front Neurol. 2025 Mar 19;16:1555630. doi: 10.3389/fneur.2025.1555630 (PMC11961420; doi:10.3389/fneur.2025.1555630)
Supplement: Supplementary file 1 [file Table_1.docx]

Supplementary Table 1. Hyperparameter Tuning Ranges and Optimal Values for XGBoost

| Hyperparameter | Range | Optimal Value |
| --- | --- | --- |
| Max depth | 3-10 | 3 |
| Gamma | 0-1 | 0.00 |
| Min child weight | 1-10 | 1.00 |
| Learning rate | 0.01-0.3 | 0.01 |
| N estimators | 50-300 | 251 |
