# Supplementary figures and images for: Identifying depression with mixed features: the potential value of eye-tracking features
Source: Front Neurol. 2025 Mar 19;16:1555630. doi: 10.3389/fneur.2025.1555630 (PMC11961420; doi:10.3389/fneur.2025.1555630)

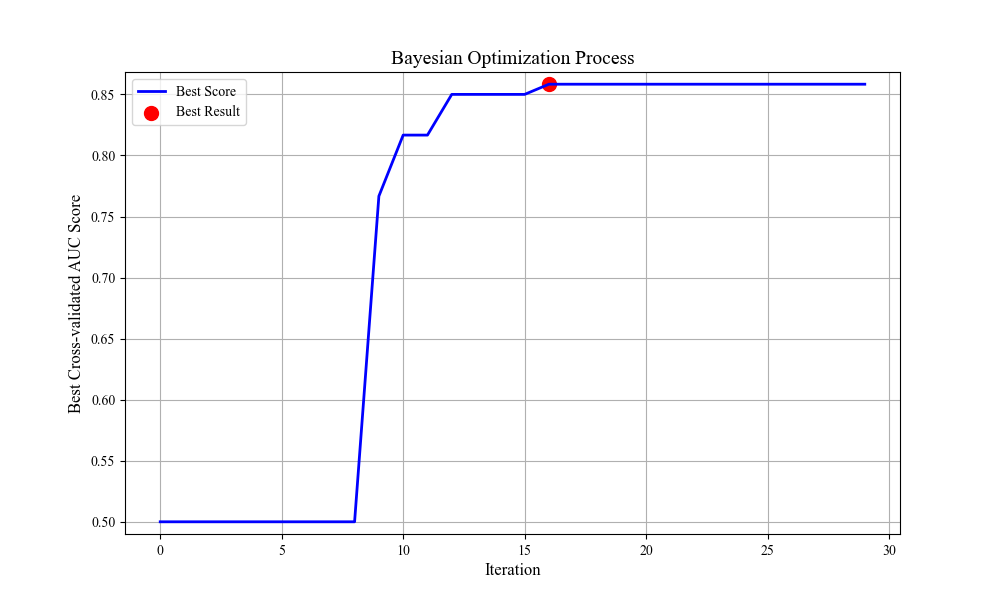

Supplement: SUPPLEMENTARY FIGURE 1 — The changes in cross-validation AUC scores during the Bayesian optimization process. As the number of iterations increases, the AUC scores gradually improve and stabilize after a certain iteration. The red dot in the figure indicates the best AUC score and the corresponding iteration number obtained throughout the optimization process. Through this optimization method, the model can iteratively adjust the hyperparameters to achieve optimal performance. [file Image_1.TIF]
